# Metabolic syndrome in patients diagnosed with affective disorders: a cross-sectional study among inpatients from two Cluj-Napoca psychiatry clinics

**DOI:** 10.3389/fpsyt.2026.1796346

**Published:** 2026-10-02

**Authors:** Patricea Iulia Huluba, Florin C. Lung, Bianca Crecan-Suciu, Ramona L. Paunescu, Anca C. Bibolar, Ioana Miclutia

**Affiliations:** Department of Neurosciences, University of Medicine & Pharmacy “Iuliu Haţieganu”, Cluj-Napoca, Romania

**Keywords:** bipolar disorder, cardiometabolic risk, cardiovascular risk, logistic regression, major depressive disorder, metabolic syndrome, psychotropic medication

## Abstract

**Background:**

Metabolic syndrome (MetS) is highly prevalent among individuals with affective disorders and contributes substantially to premature cardiovascular morbidity and mortality. However, the relative contribution of demographic characteristics, psychiatric diagnosis, and psychotropic medication to metabolic risk remains incompletely understood. This study investigated the prevalence of MetS and its associated demographic, clinical, and treatment-related factors in hospitalized patients with major depressive disorder (MDD) and bipolar disorder (BD).

**Methods:**

A cross-sectional study was conducted in 502 adult psychiatric inpatients diagnosed with MDD or BD. Metabolic syndrome was defined according to the National Cholesterol Education Program Adult Treatment Panel III (NCEP ATP III) criteria. Demographic characteristics, psychiatric diagnosis, metabolic parameters, and psychotropic medication were analyzed. Independent correlates of MetS were identified using multivariable binary logistic regression, excluding variables constituting the diagnostic criteria for MetS to avoid circularity.

**Results:**

Metabolic syndrome was identified in 25.4% of the study population. Patients with MetS were significantly older and more frequently female than those without MetS (both p < 0.001). Although bipolar disorder was not significantly associated with MetS in univariate analyses, it became independently associated with MetS after multivariable adjustment (adjusted OR 1.77, 95% CI 1.04–3.02; p = 0.037), together with increasing age (adjusted OR 1.06 per year, 95% CI 1.04–1.08; p < 0.001) and female sex (adjusted OR 1.87, 95% CI 1.18–2.98; p = 0.008). Current psychotropic medication, assessed at the time of hospital admission, was not independently associated with MetS after adjustment for demographic and clinical characteristics. These findings should not be interpreted as evidence against the established long-term metabolic effects of psychotropic medication.

**Conclusions:**

Metabolic syndrome represents a common medical comorbidity among hospitalized patients with affective disorders. Increasing age, female sex, and bipolar disorder were independently associated with MetS, whereas current psychotropic medication, assessed cross-sectionally at hospital admission, was not independently associated with metabolic syndrome after adjustment. This finding should be interpreted in the context of unavailable information regarding cumulative treatment exposure, duration, dosage, and previous medication history. These findings support routine metabolic monitoring as an integral component of psychiatric care, particularly among older adults, women, and patients with bipolar disorder.

## 1 Introduction

Metabolic syndrome (MetS) is a cluster of interconnected metabolic abnormalities, including abdominal obesity, hypertension, hyperglycemia, hypertriglyceridemia, and reduced high-density lipoprotein cholesterol (HDL-C), that markedly increase the risk of cardiovascular disease, type 2 diabetes mellitus, and premature mortality (1, 2). The worldwide prevalence of MetS has increased considerably during the last decades, making it one of the leading public health challenges because of its association with cardiovascular morbidity, reduced quality of life, and increased healthcare costs (2, 3).

Patients diagnosed with affective disorders, particularly major depressive disorder (MDD) and bipolar disorder (BD), are especially vulnerable to developing metabolic syndrome. The association between affective disorders and metabolic dysfunction is complex and bidirectional. Depression has been identified as an independent risk factor for the development of MetS, while metabolic abnormalities may also increase the risk of depressive symptoms through shared biological mechanisms, including chronic low-grade inflammation, hypothalamic-pituitary-adrenal (HPA) axis dysregulation, oxidative stress, insulin resistance, and alterations in adipokine signaling (4–6). Similar mechanisms have been proposed for bipolar disorder, where recurrent mood episodes, inflammatory activation, mitochondrial dysfunction, and neuroendocrine alterations contribute to metabolic impairment (7, 8).

Besides these biological mechanisms, several behavioral and treatment-related factors further increase metabolic risk in psychiatric populations. Reduced physical activity, unhealthy dietary habits, smoking, alcohol consumption, sleep disturbances, and socioeconomic disadvantage are frequently encountered in patients with mood disorders and contribute substantially to the development of obesity and insulin resistance (9). In addition, psychotropic medications, particularly second-generation antipsychotics such as clozapine and olanzapine, as well as several mood stabilizers and antidepressants, have been consistently associated with weight gain, dyslipidemia, impaired glucose metabolism, and an increased risk of metabolic syndrome (10, 11). Consequently, current international guidelines recommend routine metabolic monitoring in patients receiving long-term psychopharmacological treatment (12).

Previous studies have consistently demonstrated that the prevalence of MetS is considerably higher among patients with affective disorders than in the general population (13). Meta-analyses have reported particularly high prevalence rates among patients with bipolar disorder, whereas patients with major depressive disorder also exhibit substantially increased metabolic risk (13, 14). Female sex, older age, obesity, hypertension, diabetes mellitus, dyslipidemia, and exposure to psychotropic medication have repeatedly been identified as important factors associated with MetS (10, 13–15). Nevertheless, the reported prevalence varies considerably across studies because of differences in diagnostic criteria, patient characteristics, ethnicity, healthcare settings, and treatment exposure.

Although the relationship between affective disorders and metabolic syndrome has been extensively investigated, data from Eastern European psychiatric populations remain limited. Furthermore, relatively few studies have simultaneously compared hospitalized patients with MDD and BD while evaluating demographic and metabolic factors associated with MetS within the same clinical cohort. Such information is clinically relevant because early identification of high-risk patients may facilitate targeted preventive interventions and reduce long-term cardiovascular morbidity.

Therefore, the primary objective of the present study was to determine the prevalence of metabolic syndrome among hospitalized patients diagnosed with major depressive disorder and bipolar disorder in two psychiatric clinics from Cluj-Napoca, Romania. The secondary objectives were to compare the prevalence of MetS between diagnostic groups and to identify demographic, clinical, and treatment-related factors associated with MetS.

## 2 Materials and methods

### 2.1 Study design and participants

This cross-sectional study included 502 consecutive adult inpatients admitted to the First and Second Psychiatry Clinics of the Emergency Clinical County Hospital Cluj-Napoca, Romania, between January 1, 2020, and March 31, 2023.

The study population comprised 374 patients diagnosed with Major Depressive Disorder (MDD) and 128 patients diagnosed with Bipolar Disorder (BD). Psychiatric diagnoses were established according to the Diagnostic and Statistical Manual of Mental Disorders, Fifth Edition (DSM-5) criteria.

Eligible participants were adults (≥18 years) with a confirmed diagnosis of MDD or BD who had complete anthropometric, clinical, and laboratory data required for the assessment of metabolic syndrome. Patients with substance use disorders, dementia or other major neurocognitive disorders, severe neurological diseases, or incomplete metabolic data were excluded.

The study protocol was approved by the Ethics Committee of the “Iuliu Haţieganu” University of Medicine and Pharmacy, Cluj-Napoca, Romania (Approval No. 352/19.11.2020). All participants provided written informed consent before enrollment, and all procedures were conducted in accordance with the Declaration of Helsinki.

### 2.2 Psychiatric assessment

The diagnosis of MDD and BD was established according to the DSM-5 diagnostic criteria.

### 2.3 Clinical, laboratory and treatment variables

Demographic variables included age, sex, and place of residence (urban or rural).

Clinical assessment included waist circumference, systolic and diastolic blood pressure, history of hypertension, diabetes mellitus, and anthropometric measurements performed during hospitalization.

Laboratory investigations included fasting serum glucose, triglycerides, and high-density lipoprotein cholesterol (HDL-C), obtained from venous blood samples collected after an overnight fast according to the hospital’s standard laboratory procedures.

Information regarding psychotropic treatment at the time of hospital admission was extracted from the patients’ medical records. Medications were classified into the following therapeutic groups: antidepressants; first-generation antipsychotics; second-generation antipsychotics; mood stabilizers; anxiolytics/hypnotics; therapeutic-class polypharmacy (≥3 psychotropic classes).

These variables were collected to characterize treatment patterns within the study population and to explore their potential association with metabolic syndrome.

### 2.4 Definition of metabolic syndrome

Metabolic syndrome was defined according to the National Cholesterol Education Program Adult Treatment Panel III (NCEP ATP III) criteria.

Participants were considered to have metabolic syndrome when at least three of the following criteria were present:

- waist circumference ≥102 cm in men or ≥88 cm in women;
- triglycerides ≥150 mg/dL (1.7 mmol/L) or treatment for hypertriglyceridemia;
- HDL cholesterol <40 mg/dL in men or <50 mg/dL in women or treatment for reduced HDL cholesterol;
- blood pressure ≥130/85 mmHg or current antihypertensive treatment;
- fasting plasma glucose ≥100 mg/dL or previously diagnosed diabetes mellitus or current antidiabetic treatment.

### 2.5 Statistical analysis

All statistical analyses were performed using IBM SPSS Statistics for Windows, version 26.0 (IBM Corp., Armonk, NY, USA).

Continuous variables were expressed as mean ± standard deviation (SD) or median with interquartile range (IQR), depending on data distribution, whereas categorical variables were presented as frequencies and percentages.

The normality of continuous variables was assessed using the Kolmogorov–Smirnov test and graphical inspection of histograms and Q–Q plots.

Comparisons between two independent groups were performed using the independent samples t-test or Welch’s t-test when homogeneity of variances was violated. Associations between categorical variables were evaluated using Pearson’s chi-square test, and odds ratios (ORs) with 95% confidence intervals (95% CI) were calculated where appropriate.

Binary logistic regression analysis was performed to identify variables independently associated with metabolic syndrome. The dependent variable was the presence of metabolic syndrome (yes/no). Based on clinical relevance and the results of the univariate analyses, age, sex, psychiatric diagnosis (major depressive disorder vs. bipolar disorder), place of residence, second-generation antipsychotic exposure, and therapeutic-class polypharmacy were entered into the final model. The selection of covariates was prespecified to identify independent correlates of metabolic syndrome while minimizing overfitting. Variables constituting the NCEP ATP III definition of metabolic syndrome (abdominal obesity, hypertension, fasting glucose, triglycerides, HDL cholesterol, and diabetes mellitus) were intentionally excluded from the regression model to avoid circularity. Adjusted odds ratios (aORs) with 95% confidence intervals (95% CIs) were reported.

Metabolic syndrome status could not be determined for five participants because of incomplete metabolic data. These participants were excluded only from analyses requiring MetS classification, while all other available data were retained for descriptive analyses.

All statistical tests were two-sided, and a p-value <0.05 was considered statistically significant.

## 3 Results

### 3.1 Study population

A total of 502 consecutive adult inpatients diagnosed with an affective disorder were included in the study. The study population comprised 374 patients with Major Depressive Disorder (MDD) and 128 patients with Bipolar Disorder (BD).

Metabolic syndrome (MetS) status could be unequivocally determined for 497 participants. Five patients presented incomplete metabolic data that prevented definitive classification according to the NCEP ATP III criteria and were therefore excluded from analyses involving MetS. The study flow is presented in **Figure 1**.

**Figure 1:**
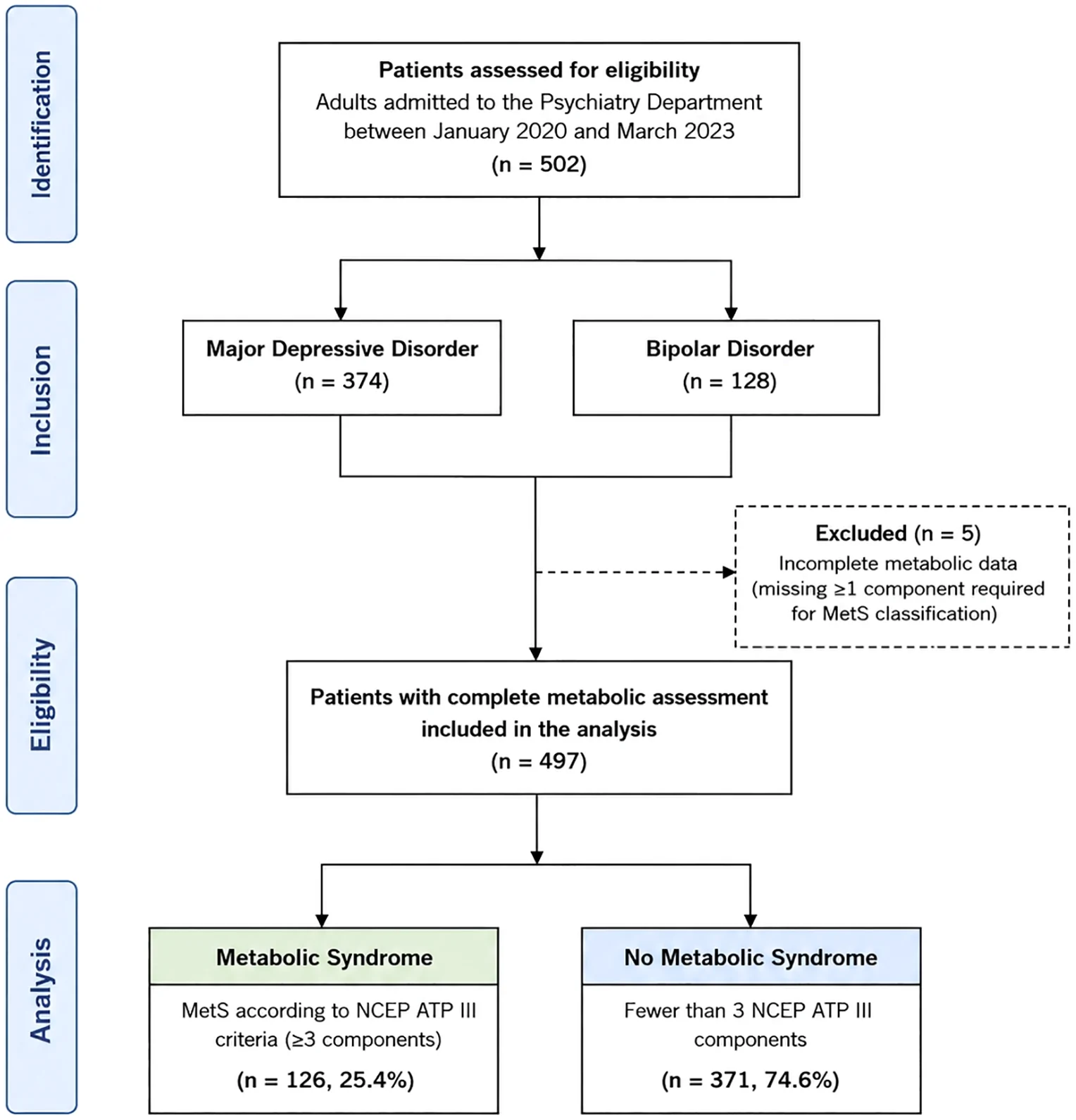
STROBE flow diagram of the study population. MetS, metabolic syndrome; NCEP ATP III, National Cholesterol Education Program Adult Treatment Panel III.

Among the 497 evaluable participants, 126 fulfilled at least three NCEP ATP III criteria, corresponding to an overall prevalence of 25.4%.

### 3.2 Baseline characteristics of the study population

The final study population consisted of 497 evaluable participants, including 369 patients with major depressive disorder (MDD) and 128 patients with bipolar disorder (BD). The overall mean age was 49.1 ± 15.3 years, and women accounted for 56.1% of the study population (**Table 1**).

**Table 1:** Baseline characteristics according to psychiatric diagnosis (MDD, major depressive disorder; BD, bipolar disorder; SD, standard deviation; HDL-C, high-density lipoprotein cholesterol; IQR, interquartile range).

Variable	Overall (N = 497)	MDD (n=369)	BD (n=128)	Test statistic	P
Age, years, mean ± SD	49.07 ± 15.28	48.98 ± 15.63	49.33 ± 14.29	-0.233	0.816
Female sex	279 (56.1%)	220 (59.6%)	59 (46.1%)	χ²=7.062	0.008
Urban residence	317 (63.8%)	233 (63.1%)	84 (65.6%)	χ²=0.253	0.615
Hypertension	171 (34.4%)	124 (33.6%)	47 (36.7%)	χ²=0.408	0.523
Diabetes mellitus	63 (12.7%)	39 (10.6%)	24 (18.8%)	χ²=5.746	0.017
Abdominal obesity	126 (25.4%)	83 (22.5%)	43 (33.6%)	χ²=6.188	0.013
Metabolic syndrome	126 (25.4%)	88 (23.8%)	38 (29.7%)	χ²=1.712	0.191
Any antidepressant	385 (77.5%)	354 (95.9%)	31 (24.2%)	χ²=279.994	<0.001
Second-generation antipsychotic	291 (58.6%)	177 (48.0%)	114 (89.1%)	χ²=66.132	<0.001
First-generation antipsychotic	66 (13.3%)	22 (6.0%)	44 (34.4%)	χ²=66.620	<0.001
Mood stabilizer	262 (52.7%)	151 (40.9%)	111 (86.7%)	χ²=79.966	<0.001
Z-drug	184 (37.0%)	164 (44.4%)	20 (15.6%)	χ²=33.853	<0.001
Therapeutic-class polypharmacy (≥3)	245 (49.3%)	185 (50.1%)	60 (46.9%)	χ²=0.404	0.525
Triglycerides, mg/dL, median (IQR)	116.0 (87.0–167.0)	117.0 (88.0–167.0)	112.0 (84.0–164.5)	U=21721.5	0.356
HDL-C, mg/dL, median (IQR)	50.0 (41.0–60.0)	50.0 (42.0–61.0)	49.0 (39.0–57.0)	U=19483.0	0.127
Fasting glucose, mg/dL, median (IQR)	86.5 (80.0–100.0)	86.0 (79.0–100.0)	89.0 (81.0–101.2)	U=25759.5	0.114

Age did not differ significantly between patients with MDD and BD (48.98 ± 15.63 vs. 49.33 ± 14.29 years, p = 0.816). Female sex was significantly more frequent among patients with MDD than among those with BD (59.6% vs. 46.1%, p = 0.008), whereas the proportion of patients from urban areas was comparable between diagnostic groups (63.1% vs. 65.6%, p = 0.615). Diabetes mellitus (18.8% vs. 10.6%, p = 0.017) and abdominal obesity (33.6% vs. 22.5%, p = 0.013) were more common among patients with BD, whereas hypertension did not differ significantly between groups (36.7% vs. 33.6%, p = 0.523).

The overall prevalence of metabolic syndrome in the study population was 25.4%, corresponding to 23.8% among patients with MDD and 29.7% among patients with BD. Metabolic syndrome was numerically more frequent among patients with BD than among those with MDD (29.7% vs. 23.8%); however, this difference did not reach statistical significance (p = 0.191). Median triglyceride, HDL-C, and fasting glucose concentrations did not differ significantly between patients with MDD and BD (all p > 0.05).

As expected, pharmacological treatment differed substantially between diagnostic groups. Patients with BD more frequently received mood stabilizers, first-generation antipsychotics, and second-generation antipsychotics, whereas antidepressants and Z-drugs were prescribed predominantly in patients with MDD (all p < 0.001). Therapeutic-class polypharmacy did not differ significantly between diagnostic groups (p = 0.525).

### 3.3 Clinical factors associated with metabolic syndrome

The comparison between patients with and without metabolic syndrome is presented in **Table 2**.

**Table 2:** Comparison between patients with and without metabolic syndrome.

Variable	MetS (n=126)	No MetS (n=371)	OR (95% CI)	Test statistic	P
Age, years, mean ± SD	57.65 ± 11.03	46.15 ± 15.44	—	Welch t=9.068	<0.001
Female sex	87 (69.0%)	192 (51.8%)	2.08 (1.35–3.19)	χ²=11.426	<0.001
Bipolar disorder	38 (30.2%)	90 (24.3%)	1.35 (0.86–2.11)	χ²=1.712	0.191
Urban residence	75 (59.5%)	242 (65.2%)	0.78 (0.52–1.19)	χ²=1.325	0.250
Hypertension	101 (80.2%)	70 (18.9%)	17.37 (10.44–28.91)	χ²=156.559	<0.001
Diabetes mellitus	50 (39.7%)	13 (3.5%)	18.12 (9.38–35.00)	χ²=111.217	<0.001
Abdominal obesity	84 (66.7%)	42 (11.3%)	15.67 (9.60–25.58)	χ²=152.240	<0.001
Triglycerides, mg/dL, median (IQR)	172.0 (135.0–217.0)	107.0 (80.8–140.2)	—	U=35986.0	<0.001
HDL-C, mg/dL, median (IQR)	44.0 (37.0–55.0)	52.0 (43.0–61.0)	—	U=15578.0	<0.001
Fasting glucose, mg/dL, median (IQR)	105.5 (86.0–126.2)	84.0 (78.2–93.0)	—	U=34961.0	<0.001

Data are presented as mean ± standard deviation, median (interquartile range), or number (percentage). Odds ratios are derived from univariate analyses of categorical variables only. Continuous metabolic parameters are presented descriptively because they constitute diagnostic components of metabolic syndrome and were not interpreted as independent risk factors. (MetS, metabolic syndrome; OR, odds ratio; CI, confidence interval; SD, standard deviation; IQR, interquartile range; HDL-C, high-density lipoprotein cholesterol).

The demographic and clinical characteristics of patients according to metabolic syndrome status are presented in **Table 2**. Patients with MetS were significantly older than those without MetS (57.65 ± 11.03 vs. 46.15 ± 15.44 years, p < 0.001). Female sex was significantly associated with MetS, with women presenting approximately two-fold higher unadjusted odds than men (OR 2.08, 95% CI 1.35–3.19; p < 0.001). Although bipolar disorder was numerically more frequent among patients with MetS (30.2% vs. 24.3%), this difference did not reach statistical significance (OR 1.35, 95% CI 0.86–2.11; p = 0.191). Similarly, place of residence was not associated with metabolic syndrome (p = 0.250).

As expected, hypertension, diabetes mellitus, and abdominal obesity were markedly more prevalent among patients with MetS (all p < 0.001). These variables constitute diagnostic components of the NCEP ATP III definition and are therefore presented descriptively rather than interpreted as independent predictors of the syndrome. Likewise, patients with MetS had significantly higher fasting glucose and triglyceride concentrations and lower HDL-C levels than patients without MetS (all p < 0.001).

### 3.4 Psychotropic treatment and metabolic syndrome

Psychotropic medication use at the time of hospitalization is summarized in **Table 3**. Because treatment allocation reflected clinical judgment and diagnosis rather than random assignment, these analyses should be interpreted as descriptive and exploratory.

**Table 3:** Psychotropic medication according to metabolic syndrome status.

Medication class	MetS (n=126)	No MetS (n=371)	OR (95% CI)	Test statistic	P
Any antidepressant	95 (75.4%)	290 (78.2%)	0.86 (0.53–1.38)	χ²=0.413	0.520
SSRI	53 (42.1%)	167 (45.0%)	0.89 (0.59–1.33)	χ²=0.332	0.565
SNRI	27 (21.4%)	71 (19.1%)	1.15 (0.70–1.90)	χ²=0.312	0.577
Tricyclic antidepressant	0 (0.0%)	3 (0.8%)	NE	χ²=1.025	0.575
Mirtazapine	24 (19.0%)	60 (16.2%)	1.22 (0.72–2.06)	χ²=0.554	0.457
Trazodone	17 (13.5%)	44 (11.9%)	1.16 (0.64–2.11)	χ²=0.233	0.630
Other antidepressant	9 (7.1%)	20 (5.4%)	1.35 (0.60–3.05)	χ²=0.525	0.469
Second-generation antipsychotic	67 (53.2%)	224 (60.4%)	0.75 (0.50–1.12)	χ²=2.011	0.156
First-generation antipsychotic	19 (15.1%)	47 (12.7%)	1.22 (0.69–2.18)	χ²=0.475	0.491
Olanzapine/clozapine exposure	5 (4.0%)	66 (17.8%)	0.19 (0.08–0.49)	χ²=14.674	<0.001
Any mood stabilizer	53 (42.1%)	209 (56.3%)	0.56 (0.37–0.85)	χ²=7.685	0.006
Lithium	0 (0.0%)	4 (1.1%)	NE	χ²=1.370	0.576
Valproate	44 (34.9%)	167 (45.0%)	0.66 (0.43–1.00)	χ²=3.922	0.048
Carbamazepine	8 (6.3%)	39 (10.5%)	0.58 (0.26–1.27)	χ²=1.904	0.168
Lamotrigine	1 (0.8%)	4 (1.1%)	0.73 (0.08–6.63)	χ²=0.076	1.000
Benzodiazepine	0 (0.0%)	0 (0.0%)	NE	Fisher exact	
Z-drug	47 (37.3%)	137 (36.9%)	1.02 (0.67–1.54)	χ²=0.006	0.940
Buspirone	8 (6.3%)	29 (7.8%)	0.80 (0.36–1.80)	χ²=0.294	0.588
Gabapentinoid	23 (18.3%)	39 (10.5%)	1.90 (1.09–3.33)	χ²=5.163	0.023
Therapeutic-class polypharmacy (≥3)	51 (40.5%)	194 (52.3%)	0.62 (0.41–0.93)	χ²=5.253	0.022

Data are presented as number (percentage). Therapeutic-class polypharmacy was defined as the concurrent use of three or more psychotropic medication classes. P values were calculated using Pearson’s χ² test or Fisher’s exact test, as appropriate. Medication exposure was assessed at the time of hospital admission and reflects current treatment rather than cumulative lifetime exposure.

Antidepressants were the most frequently prescribed psychotropic medications in the overall cohort, followed by second-generation antipsychotics and mood stabilizers. The distribution of individual therapeutic classes differed according to psychiatric diagnosis, reflecting current clinical practice in the management of affective disorders.

In the univariate analyses, no significant associations were observed between metabolic syndrome and the use of any antidepressant, selective serotonin reuptake inhibitors (SSRIs), serotonin-norepinephrine reuptake inhibitors (SNRIs), mirtazapine, trazodone, first-generation antipsychotics, second-generation antipsychotics, or benzodiazepines (all p > 0.05).

Mood stabilizer treatment, valproate exposure, gabapentinoid use, and therapeutic-class polypharmacy differed significantly between patients with and without metabolic syndrome (**Table 3**). However, these univariate associations should be interpreted with caution because psychotropic treatment was recorded only at the time of hospital admission and primarily reflected clinical indication and psychiatric diagnosis rather than random treatment allocation. Consequently, these findings do not imply a causal relationship between individual medications and metabolic syndrome.

Exposure to olanzapine or clozapine also differed between groups. Given the limited number of exposed patients and the absence of information regarding treatment duration, cumulative dose, or previous medication history, this finding was considered exploratory and was not interpreted as evidence of a protective or causal effect.

Because medication exposure was evaluated cross-sectionally and information regarding cumulative dose, treatment duration, previous medication switches, and lifetime exposure was unavailable, psychotropic treatment was entered into the multivariable model only as clinically meaningful therapeutic categories. This strategy reduced model complexity while allowing adjustment for the potential influence of current pharmacological treatment on metabolic syndrome.

### 3.5 Multivariable logistic regression analysis

To identify variables independently associated with metabolic syndrome while avoiding circularity, a multivariable binary logistic regression model was constructed including only variables that were not part of the NCEP ATP III diagnostic criteria. The final model included age, sex, psychiatric diagnosis, place of residence, second-generation antipsychotic exposure, and therapeutic-class polypharmacy (**Table 4**).

**Table 4:** Multivariable logistic regression analysis identifying independent correlates of metabolic syndrome.

Predictor	B	S.E.	Wald	df	p	Exp(B)	95% CI
Constant	-4.191	0.554	57.278	1	<0.001	0.015	0.005–0.045
Age (per year)	0.056	0.009	42.361	1	<0.001	1.058	1.040–1.076
Female sex	0.627	0.236	7.042	1	0.008	1.872	1.178–2.976
Bipolar disorder	0.571	0.273	4.369	1	0.037	1.770	1.036–3.024
Urban residence	-0.185	0.230	0.644	1	0.422	0.831	0.529–1.305
Second-generation antipsychotic	-0.302	0.257	1.380	1	0.240	0.739	0.447–1.224
Polypharmacy (≥3 classes)	-0.155	0.238	0.424	1	0.515	0.857	0.537–1.365

OR, odds ratio; CI, confidence interval; S.E., standard error.

Variables constituting the diagnostic criteria for metabolic syndrome (abdominal obesity, fasting glucose, triglycerides, HDL cholesterol, and hypertension) were intentionally excluded from the regression analysis because they are integral components of the outcome definition and their inclusion would have resulted in circular modeling.

Increasing age remained independently associated with metabolic syndrome after adjustment for the other covariates. Each additional year of age increased the odds of metabolic syndrome by approximately 5.8% (adjusted OR 1.058, 95% CI 1.040–1.076; p < 0.001). Female sex also remained an independent predictor, with women presenting significantly higher odds of metabolic syndrome than men (adjusted OR 1.872, 95% CI 1.178–2.976; p = 0.008).

Patients with bipolar disorder showed significantly higher adjusted odds of metabolic syndrome compared with patients diagnosed with major depressive disorder (adjusted OR 1.770, 95% CI 1.036–3.024; p = 0.037), despite the absence of a statistically significant association in the univariate analysis. This pattern is consistent with negative confounding in the univariate analysis. Simultaneous adjustment for age, sex, place of residence, second-generation antipsychotic exposure, and therapeutic-class polypharmacy reduced the influence of these confounding variables and revealed an independent association between bipolar disorder and metabolic syndrome. Because the objective of the regression analysis was to identify independent correlates rather than to evaluate sequential changes in effect estimates across nested models, a single prespecified multivariable model was retained.

Urban residence, second-generation antipsychotic exposure, and therapeutic-class polypharmacy were not independently associated with metabolic syndrome after multivariable adjustment (all p > 0.05).

These findings indicate that demographic characteristics, particularly age and female sex, showed stronger independent associations with metabolic syndrome than current psychotropic medication exposure assessed at hospital admission. Because cumulative treatment history was unavailable, these findings should not be interpreted as evidence against the established long-term metabolic effects of psychotropic medication.

## 4 Discussion

The present study investigated the prevalence of metabolic syndrome (MetS) and its associated demographic, clinical, and treatment-related factors in a cohort of hospitalized patients diagnosed with major depressive disorder (MDD) and bipolar disorder (BD). Three principal findings emerged from the present analysis. First, approximately one quarter of the study population fulfilled the diagnostic criteria for MetS, confirming the substantial metabolic burden associated with affective disorders. Second, increasing age, female sex, and bipolar disorder remained independently associated with MetS after adjustment for potential confounding variables. Third, although several psychotropic medication classes demonstrated significant associations in univariate analyses, these associations disappeared after multivariable adjustment, suggesting that the development of metabolic syndrome is influenced by a complex interaction between demographic characteristics, psychiatric diagnosis, cumulative disease burden, and pharmacological treatment rather than current medication exposure alone.

The overall prevalence of metabolic syndrome observed in our cohort (25.4%) is consistent with previous reports indicating that patients with affective disorders experience a markedly higher burden of cardiometabolic disease than the general population (16–18). Large systematic reviews and meta-analyses have consistently demonstrated that both MDD and BD are associated with increased rates of obesity, insulin resistance, dyslipidemia, hypertension, and type 2 diabetes mellitus, all of which contribute substantially to premature cardiovascular mortality (17–20). Importantly, cardiovascular disease remains one of the leading causes of excess mortality among patients with severe mental illness, reducing life expectancy by approximately 10–20 years compared with the general population (16, 18).

Although the prevalence of metabolic syndrome in our cohort was slightly lower than the pooled estimates reported in several international meta-analyses, our findings remain well within the range described in previous studies. Reported prevalence estimates vary considerably depending on the diagnostic criteria applied, ethnicity, healthcare system, study setting, and psychotropic prescribing practices (17, 19). In particular, studies including chronically hospitalized psychiatric populations generally report higher prevalence rates than investigations performed in outpatient settings, reflecting greater illness severity, longer disease duration, and more prolonged exposure to psychotropic medication (18, 21). Conversely, studies involving first-episode or drug-naïve patients usually report substantially lower frequencies of metabolic abnormalities (17).

An important strength of the present investigation is that it provides contemporary data from an Eastern European psychiatric population. Most epidemiological studies evaluating metabolic syndrome in affective disorders originate from North America, Western Europe, or East Asia, whereas relatively limited evidence is available from Central and Eastern Europe. Consequently, our findings contribute additional information regarding the cardiometabolic profile of Romanian psychiatric inpatients treated within routine clinical practice. Differences in dietary habits, socioeconomic status, accessibility of healthcare services, and prescribing strategies may partly explain regional variability in the prevalence of metabolic syndrome and further highlight the importance of locally generated epidemiological data (18, 22).

Age emerged as the strongest independent predictor of metabolic syndrome in the multivariable analysis. This finding is highly consistent with previous epidemiological studies demonstrating that metabolic risk progressively increases throughout adulthood owing to cumulative exposure to visceral adiposity, insulin resistance, chronic low-grade inflammation, endothelial dysfunction, oxidative stress, and endocrine alterations (19, 23, 24). Beyond physiological aging, patients affected by mood disorders frequently accumulate additional cardiometabolic risk factors during the course of illness, including recurrent affective episodes, prolonged sedentary behavior, poor dietary habits, smoking, sleep disturbances, and long-term pharmacological treatment (25). These mechanisms probably act synergistically, accelerating metabolic deterioration and increasing cardiovascular risk (18, 20, 24).

Place of residence was included in the multivariable model because differences in lifestyle, healthcare access, and socioeconomic characteristics between urban and rural populations could potentially influence metabolic risk. However, no independent association with metabolic syndrome was observed in the present cohort, suggesting that residential setting did not contribute substantially to metabolic risk after adjustment for the other covariates.

Female sex was independently associated with nearly two-fold higher odds of metabolic syndrome in our study. Although previous investigations have reported heterogeneous findings regarding sex-specific vulnerability, several recent studies support an increased prevalence of metabolic syndrome among women diagnosed with affective disorders, particularly after menopause (21, 26). The underlying mechanisms are likely multifactorial. Declining estrogen concentrations promote visceral fat accumulation, insulin resistance, endothelial dysfunction, and chronic inflammation, all of which contribute to the development of metabolic syndrome (27). Moreover, women with mood disorders may present greater inflammatory activation, altered adipokine secretion, reduced physical activity during depressive episodes, and increased susceptibility to psychotropic-induced weight gain compared with men (21, 27, 28).

One of the most relevant findings of the present study is that bipolar disorder became independently associated with metabolic syndrome after multivariable adjustment, despite the absence of a statistically significant association in the univariate analysis. This pattern is consistent with negative confounding, indicating that adjustment for demographic and treatment-related variables revealed an independent association that was not apparent in the crude analysis. Although previous studies have consistently reported a greater prevalence of metabolic syndrome among patients with bipolar disorder than among those with major depressive disorder, the mechanisms underlying this association remain incompletely understood (18, 29, 30). Our findings support the hypothesis that bipolar disorder should no longer be regarded exclusively as a disorder of mood regulation but rather as a multisystem disease involving profound metabolic, endocrine, inflammatory, and circadian disturbances.

The mechanisms underlying the association between bipolar disorder and metabolic syndrome are likely multifactorial (31). Current evidence suggests that chronic low-grade inflammation, hypothalamic–pituitary–adrenal axis dysregulation, circadian rhythm disturbances, and adverse lifestyle factors all contribute to increased cardiometabolic vulnerability in bipolar disorder. Rather than acting independently, these biological and behavioral mechanisms probably interact throughout the course of illness, ultimately increasing the long-term risk of metabolic syndrome and cardiovascular disease (17, 29, 30, 32–38).

The relationship between psychotropic medication and metabolic syndrome deserves careful interpretation. Although several medication classes demonstrated statistically significant associations in the univariate analyses, these associations disappeared after multivariable adjustment. This finding should not be interpreted as evidence that psychotropic medications are metabolically neutral. Rather, our results indicate only that current medication exposure was not independently associated with metabolic syndrome after adjustment within this cross-sectional cohort and should not be extrapolated to cumulative lifetime treatment effects. Instead, it most likely reflects the complexity of treatment allocation in routine clinical practice. Medication choice is determined by psychiatric diagnosis, illness severity, treatment resistance, previous therapeutic response, psychiatric comorbidity, and clinician preference, creating substantial confounding by indication. Consequently, patients receiving certain medications often differ systematically from those receiving alternative treatments, making causal interpretation difficult in cross-sectional analyses.

Furthermore, our study evaluated psychotropic treatment only at the time of hospital admission. We were unable to account for cumulative lifetime exposure, duration of treatment, dosage, previous medication switches, or adherence, all of which substantially influence long-term metabolic outcomes (39, 40). Numerous longitudinal studies continue to demonstrate that second-generation antipsychotics—particularly olanzapine and clozapine—remain among the psychotropic agents most strongly associated with weight gain, dyslipidemia, insulin resistance, and metabolic syndrome (39–41). Similarly, certain mood stabilizers and antidepressants have been associated with variable degrees of metabolic impairment depending on treatment duration, concomitant medication, and individual patient susceptibility (39, 42). Therefore, our findings should not discourage routine metabolic monitoring during psychopharmacological treatment but rather emphasize that medication effects should always be interpreted within the broader clinical context.

The clinical implications of our findings extend beyond the identification of metabolic syndrome itself. Patients with affective disorders frequently experience premature cardiovascular mortality, which currently represents one of the leading causes of excess deaths in psychiatric populations (18, 23). Importantly, cardiovascular disease develops gradually over many years before becoming clinically apparent. Therefore, systematic screening during psychiatric follow-up offers a valuable opportunity to detect modifiable cardiometabolic risk factors at an early stage. Based on our findings, particular attention should be directed toward older adults, women, and patients diagnosed with bipolar disorder, who demonstrated the highest adjusted probability of metabolic syndrome.

Current international guidelines recommend routine evaluation of body weight, waist circumference, blood pressure, fasting plasma glucose (or glycated hemoglobin when appropriate), and lipid profile before initiation of psychotropic medication and periodically thereafter (39, 43–45). Nevertheless, several studies have shown that metabolic monitoring remains suboptimal in routine psychiatric practice despite clear guideline recommendations. Consequently, many patients develop obesity, diabetes mellitus, dyslipidemia, or hypertension before appropriate preventive measures are implemented (43–45). Our findings further support the need to integrate systematic metabolic assessment into routine psychiatric care, particularly within inpatient settings where comprehensive clinical evaluation can be performed.

The present study possesses several important strengths. First, it included a relatively large and well-characterized cohort of hospitalized patients with both major depressive disorder and bipolar disorder recruited from two tertiary psychiatric centers, reflecting routine clinical practice. Second, metabolic syndrome was defined according to internationally accepted NCEP ATP III criteria, allowing direct comparison with previous epidemiological studies. Third, detailed information regarding psychotropic medication classes enabled evaluation of treatment-related associations beyond simple diagnostic comparisons. Finally, the statistical approach combined descriptive analyses and multivariable logistic regression, providing a comprehensive assessment of demographic, clinical, and treatment-related factors associated with metabolic syndrome.

Several limitations should also be acknowledged. First, the cross-sectional design precludes conclusions regarding temporal or causal relationships between affective disorders, psychotropic treatment, and metabolic syndrome. Second, several important lifestyle variables including smoking status, alcohol consumption, dietary habits, physical activity, socioeconomic status, educational level, and family history of cardiometabolic disease, were unavailable and therefore could not be incorporated into the regression model. Third, although psychotropic medication classes were systematically recorded, information regarding cumulative treatment duration, dosage, medication adherence, previous therapeutic exposure, and treatment switching was not available. Residual confounding cannot be excluded despite multivariable adjustment because several potentially important determinants of metabolic syndrome, including smoking, dietary habits, physical activity, illness duration, and socioeconomic characteristics, were unavailable for analysis. In addition, metabolic syndrome status could not be determined for five participants because of incomplete metabolic data. Given the very small number of excluded participants (1.0% of the cohort), this is unlikely to have materially influenced the overall findings. Consequently, our analyses evaluated current medication exposure rather than lifetime pharmacological burden. Fourth, inflammatory biomarkers, endocrine parameters, insulin resistance indices, and genetic or metabolomic markers were unavailable, preventing a more detailed investigation of the biological mechanisms linking affective disorders with metabolic syndrome. Also, because all participants were recruited from psychiatric inpatient units in Romania, the generalizability of the present findings to outpatient populations or other healthcare systems should be interpreted with appropriate caution. Finally, the diagnostic groups were unequal in size, with substantially more patients diagnosed with major depressive disorder than bipolar disorder. Although multivariable adjustment was performed, this imbalance may have reduced statistical precision for diagnosis-specific estimates.

Importantly, these limitations should not diminish the clinical relevance of the present findings. Instead, they identify several priorities for future research. Prospective longitudinal studies integrating repeated metabolic assessments, cumulative psychotropic exposure, inflammatory biomarkers, endocrine profiling, lifestyle characteristics, and objective measures of physical activity will be essential for clarifying the temporal relationship between psychiatric illness and metabolic dysfunction. Such multidimensional approaches may facilitate earlier identification of high-risk patients and support the development of personalized preventive strategies capable of improving both psychiatric and cardiovascular outcomes.

Taken together, our findings reinforce the need for integrated metabolic assessment as a routine component of psychiatric care, particularly among older adults, women, and patients with bipolar disorder.

## 5 Conclusion

Metabolic syndrome represents a common medical comorbidity among hospitalized patients with affective disorders, affecting 25.4% of the study population. Increasing age, female sex, and bipolar disorder were independently associated with metabolic syndrome after multivariable adjustment, whereas current psychotropic medication was not independently associated with metabolic syndrome after multivariable adjustment.

These findings suggest that cardiometabolic risk in affective disorders is multifactorial and cannot be attributed solely to current pharmacological treatment. Instead, it likely reflects the complex interaction between demographic characteristics, illness-related factors, cumulative metabolic burden, and environmental influences.

Routine metabolic assessment, including measurement of waist circumference, blood pressure, fasting plasma glucose, and lipid profile, should be integrated into standard psychiatric care, particularly for older adults, women, and patients with bipolar disorder. Early identification of metabolic abnormalities may facilitate timely preventive interventions, individualized treatment planning, and reduction of long-term cardiovascular morbidity.

Prospective longitudinal studies incorporating cumulative psychotropic exposure, illness duration, inflammatory and endocrine biomarkers, lifestyle characteristics, and genetic susceptibility are warranted to clarify the temporal relationship between affective disorders and metabolic dysfunction and to support the development of personalized preventive strategies.

## Data Availability

The original contributions presented in the study are included in the article/supplementary material. Further inquiries can be directed to the corresponding author.
